# Supplementary material for: Distorted body schema after mastectomy with immediate breast reconstruction: a 4-month follow up study
Source: PeerJ. 2022 Oct 3;10:e14157. doi: 10.7717/peerj.14157 (PMC9536299; doi:10.7717/peerj.14157)
Supplement: Supplemental Information 3 [file peerj-10-14157-s003.docx]

**<Exercise description>**

As the chest muscles can be shortened after breast cancer surgery, you may feel pain or discomfort during arm movements. Thus, you may feel difficulty during arm elevation, or during the weightlifting. To minimize these complaints, chest muscles stretching, and scapular exercises are recommended.

1. **Chest muscles stretching (Target muscles: pectoralis major and minor)**

As the chest muscles mobility improves, the arm movement and range of motion are increased.

| 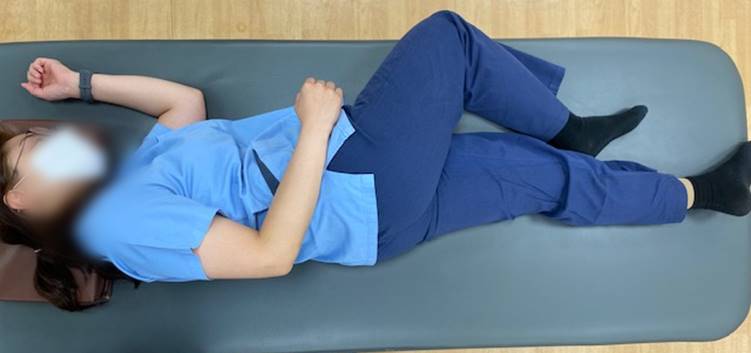 | 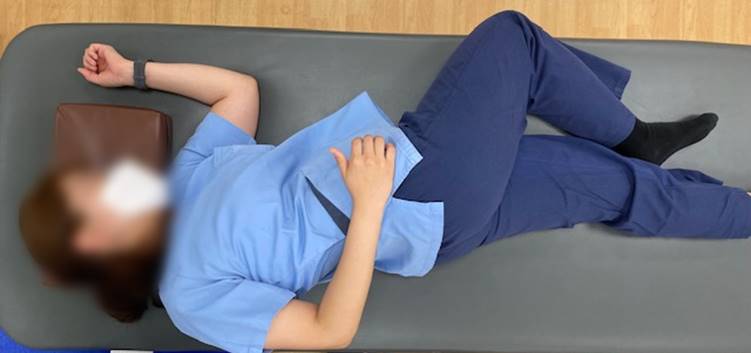 |
| --- | --- |
| 1. Start position | 1. End position |

**Example: Hold 10 seconds / Repeat 10 times / 3 sets / Twice a day / Three times a week**

1. **Lie on your sound side as (a) above pictures**
2. **Gently rotate your trunk to operated side as (b) above pictures.**
3. **Hold the position for 10 seconds and back to start position**
4. **Repeat 10 times**

**2. Scapular exercise**

You should move the scapular to backward and inward.

As the strength stabilizing the scapular to the trunk improved, the arm movement is improved.

| 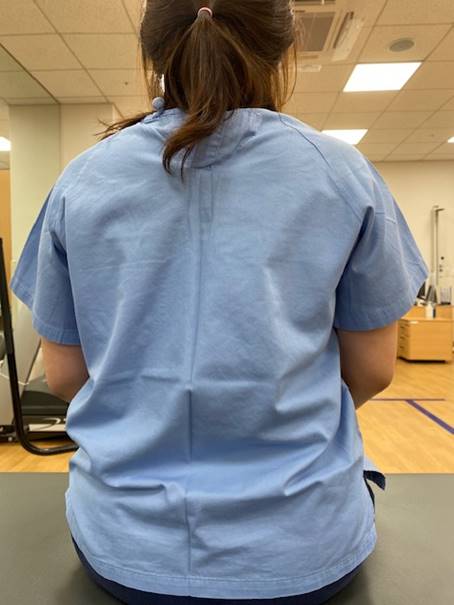 | 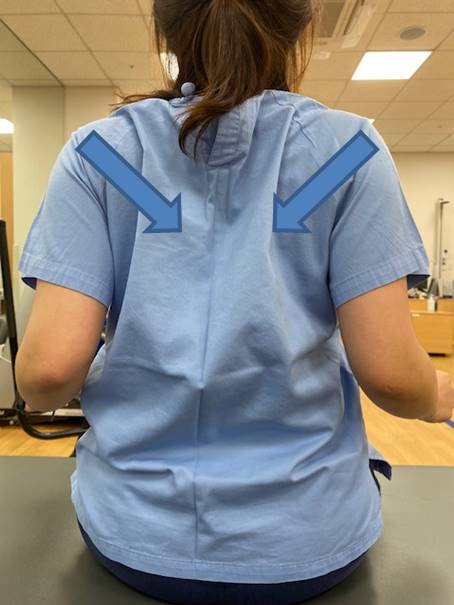 |
| --- | --- |
| 1. Start position | 1. End position |

**Example: Hold 5 seconds / Repeat 10 times / 3 sets / Twice a day / Three times a week**

1. **Sit on the chair with your feet on the ground.**
2. **Slightly bend your both elbows then squeeze both scapulars (blade bones) at back.**
3. **During the squeezing, you should feel the closeness of the two scapulars, not elbow.**

**Notice: Excessive exercise may worsen arm edema, and it also can induce shoulder muscles injury.**
